# Book Reviews

**Published:** 1824-11

**Authors:** 


### CRITICAL ANALYSIS

OF

### ENGLISH AND FOREIGN LITERATURE

RELATIVE TO THE VARIOUS BRANCHES OF

### Medical Science.

Quæ laudanda forent, et quæ culpanda, vicissim

Illa, prius, cretâ; mox hæc, carbone, *notamus.*—PERSIUS.

### DIVISION I.

### ENGLISH.

ART. I.—*Concise Narrative of an Ophthalmia, which prevailed in a Detachment of His Majesty's* 44*th Regiment, on their Voyage to Calcutta, in the Summer of* 1822; *together with an Account of other Tropical Diseases, and their Treatment on board the H. C. Ship, Warren Hastings.* By RICHARD JONES, Leamington. Fellow of the Royal College of Surgeons, and late Assistant Surgeon to the Warren Hastings, &c.—8vo. pp. 57. H. Sharpe, Warwick. 1824.

ART. II.—*Practical Remarks. Part I. on Acute and Chronic Ophthalmia, Ulcers of the Eye, &c. &c. Part II. on Remittent Fever, viz. Simple and Complicated.* By THOMAS O'HALLORAN, M.D. Author of Treatises on the Barcelona and Andalusian Yellow Fever Epidemics.—8vo. pp. 148. Burgess and Hill, London. 1824.

ART. III.—*Observations on the History and Treatment of the Ophthalmia accompanying the secondary Forms of Lues Venerea. Illustrated by Cases.* By THOMAS HEWSON, A.B. Member of the Royal College of Surgeons in Ireland; Professor of Materia Medica and Pharmacy to the College; and Surgeon to the Meath Hospital, and County of Down Infirmary, &c. &c. &c.—8vo. pp. 117. Longman and Co. 1824.

THE works which stand at the head of this article have been placed in our hands within the last few weeks; the subject upon which they principally treat is one of great importance, and, although so much has lately been written and said upon it, it appears, by the tenor of one at least of these performances, to be far from exhausted. In the days of our youth, the term ophthalmia did not convey with it impressions of so serious a nature as have of late been attached, and justly attached, to it. Since the expedition to Egypt, it has become, as it were, domesticated among us: in the army it has continued to the present day; in some of our public establishments it has obtained, and still keeps, a firm footing; and, in private life, it is not only more frequently met with, but its attacks are more formidable, and more obstinate, than heretofore. Nor is it to our own country only that these remarks are applicable; on the continent, some portions of the Prussian army have lately experienced its visitations; and, during the residence of the Army of Occupation in France, some English regiments suffered much from attacks of this malady.

It is but bare justice to say that, in the investigation of the symptoms of this disease, and in establishing a just and rational mode of treatment, the army medical officers may proudly claim a pre-eminence of merit: the work of Dr. Vetch especially, the labours of Mr. Guthrie and many others, not forgetting the late able papers of Mr. Melin, published in this Journal, have conferred high credit on their names, and a lasting benefit on posterity. Nor must we omit our unfeigned tribute of praise to the labours of Mr. Travers, whose classical performance has done much towards rescuing this disease, or rather this class of diseases, from the hands of the mere oculists; men who, however skilful they may become in the performance of operations upon this important organ, appear to us to be in general greatly ignorant of the proper constitutional treatment necessary to conduct the cure of these complaints to a happy issue.

Neither the little work of Mr. Jones, nor that of Dr. O'Halloran, are entirely devoted to the consideration of ophthalmia; but it is our intention, upon the present occasion, merely to consider this portion of their labours, and probably to notice, at some future period, the result of their observations on other diseases.

It would be unjust to close these preliminary remarks without offering our tribute of praise to the Army Medical Board, under whose auspices, and by whose judicious arrangements and regulations the professional character of the medical department of the army has attained its present high station.

We shall commence our analysis with Mr. Jones's pamphlet, only a few pages of which bear upon the present question. This gentleman sailed as assistant surgeon in the Warren Hastings, East-Indiaman, in June 1822, with a detachment of three hundred of the 44th regiment of Foot, together with their wives and children, amounting to about eighty. In about ten days after they put to sea, several of the soldiers complained of sore eyes, and a wash was made use of; but the symptoms became more urgent, and the contagious nature of the malady became apparent, says our author; "for the disease increased at once, both in the numbers whom it attacked and in malignity, assuming at length the worst forms, and every variety of ophthalmia." This paragraph of our author's requires a little consideration, since it involves the question of contagion, which, however undeniable in a certain stage of the disease, does not appear to be properly applied on the present occasion; as the sudden increase of the disease is exactly what might have been expected, where all were exposed to the same causes, (i. e. the heat of the sun by day, and the foul air of the deck at night;) whereas, a disease spreading by contagion would increase in a slower and more gradual manner. Nothing is more necessary than to have precise ideas affixed to particular terms, and in no case is this more requisite than where contagion is concerned or suspected.

Our author next proceeds to describe the symptoms of this form of ophthalmia, which usually appeared but trifling for two or three days, and then rapidly increased: it was, in fact, an acute inflammation of the conjunctiva; and the practice he adopted in the acute stage was that of general bleeding, purging, and tepid fomentation. Mr. Jones laments that he had no leeches on board: for our own parts, we greatly prefer one general bleeding in these attacks, but are ready to concede that this plan is frequently—perhaps generally, carried to a pernicious extent. In the latter stages of the disease, and to remove the opacity of the cornea, vinum opii was the common remedy.

Two cases only are detailed by our author, as illustrative of this disease; but they are, in fact, different in their nature: inasmuch as the last case was one in which inflammation had seized the internal structure of the eye, and mercury was had recourse to, with the most beneficial result. The only criticism we shall venture upon these two cases is, that in both the incipient symptoms appear to have been allowed to run on rather too long before active depletion was employed. In the first case, we are not informed how long the *trifling* inflammation of the conjunctiva had existed; but it is stated that the man was admitted on the 28th of July, that a collyrium was ordered, and a dose of sulphate of magnesia given; the next day, the palpebræ were closed, and the purulent discharge established. We shall not continue to detail the progress of the case: it is very creditable to our author, and ended favourably. In the second case, a trifling redness of the conjunctiva had existed for a week, and during that time the zinc lotion had been used, and purgatives taken, with little benefit. The result was, that inflammation spread to the sclerotica and iris. Proper measures were then resorted to; but still the administration of mercury was delayed for two days, and, although the recovery was tolerably complete, yet there was a cloudiness of the pupil and a puckering of the iris remaining, when the man left the ship.

We have thought it our duty freely to point out these circumstances to our readers, because, if there be a complaint in the catalogue of human ills that requires instant decision and prompt treatment, it is the one above mentioned. An immediate bleeding in the one case, and as immediate and rapid introduction of mercury in the other, would probably have cut short the disease in these instances.

It will presently be seen that the treatment recorded in Mr. Jones's work is not that which is recently advocated by the author next in order on our list, nor by our respected correspondent from Fort Pitt; but that point shall be considered presently, and we have only to add, in justice to Mr. Jones, that he most ingenuously gives his opinion that the earlier adoption of venesection would have cut off the disease; a fact which its subsequent adoption, in upwards of an hundred cases, fully justified.

The name of Dr. O'HALLORAN is well known to the profession as a zealous and intrepid medical officer, who has investigated, with considerable ability, the circumstances of the late fever at Barcelona, and of whose labours we had occasion to speak very favourably at no distant period. He now comes again before the public, detailing the result of his experience in ophthalmia, of which he has witnessed a great number of cases, both in Europe, America, and its islands. There will be found a very striking coincidence between this gentleman's mode of treatment and that recommended by Mr. Melin, in his very able Report from Fort Pitt; and, were it not that Dr. O'Halloran practised at Gibraltar, and the latter gentleman in England, we should almost suppose they had both been thinking and acting in concert. There are, it is true, a few shades of difference between them; but, in their general principles and views, the most striking conformity of sentiment subsists.

It will now be our business to detail Dr. O'Halloran's opinion and practice to our readers, comparing it with Mr. Melin's statement, and offering such comments as our own experience suggests; being well convinced that these gentlemen seek only the truth, and therefore will not feel disposed to quarrel with us, should we feel it our duty to dissent from them in some particular points of their practice.

Dr. O'Halloran's preface contains, in a compressed form, the substance of his opinions relative to ophthalmia, as well as of the line of practice he has latterly adopted. For twelve years he appears to have combated this disease by large and frequent bleedings, and by the strictest antiphlogistic regimen; but he was frequently disappointed when he had been almost confident of success: nay, he affirms that a review of his own practice during these twelve years proved that this disease was aggravated by low diet, depletion, confinement to bed, &c.; and it was clearly established that the exclusion of light and air was highly injurious. Under these impressions, our author visited Chatham in July of this year, and there was informed by Mr. Melin of the plan of treatment that had for the last three years been adopted by him, and which we have lately laid before our readers; a plan exactly in conformity with that which Dr. O'Halloran had employed in the 64th regiment.

Proceeding now to the work itself, we find the first chapter containing general remarks on the prevalence of acute ophthalmia in the army. That it is a disease more unmanageable in such situations than in private life, cannot for a moment be denied; a fact that is now, indeed, unfortunately established by its obstinate adherence to some of our public institutions, where, from the numbers assembled under one roof, they are placed in the same circumstances as bodies of the military. There is, indeed, something very inexplicable and difficult to solve in this perpetual renewal or propagation of the disease. We have seen it for months affecting solely one corps, out of many placed precisely in the same situation, following them in every change of quarter, in spite of every precaution of cleanliness, ventilation, &c.: nay, we have seen it persisting in only one company of a regiment, and at last disappearing suddenly, without any apparent reason.

A page or two of Dr. O'Halloran's work is occupied with severe, but not unjust, strictures upon the sarcasms to which the military surgeon has been exposed, in consequence of his being frequently baffled in his attempts to cure this disease; and with allusions to a recent investigation, which terminated so highly to the honour of the medical officers of the army, but which we do not deem it necessary to dwell upon.

Our author has described three varieties of ophthalmia: the first appears to have been a mild or subacute inflammation of the conjunctiva; the second, the more acute form of this disease; and the third variety is the purulent ophthalmia, which has usually been denominated the Egyptian. These three varieties appear to us to differ more in degree than in kind; they may be modified by both external or internal circumstances, —by the habits of the patient, as well as by his original constitution. All these three varieties may, however, so far be considered as one disease, contradistinguished from that species of affection called chronic ophthalmia. To return to our author. He describes these varieties as follows:—

"*Variety I.*—The first variety of this disease, as it appeared in the 64th regiment, exhibited redness of the conjunctiva and lining membranes of the eye-lids. The lachrymal discharge, if at all increased, was increased so inconsiderably as scarcely to deserve notice. The turgescence of the vessels of the conjunctiva and upper eye-lids was slight. It conveyed an imperfect idea of sand interposed between the membranes. The vision was little, if at all, affected. The symptoms now stated characterised the first variety. The inflammation was moderate, sufficiently active to produce redness, or what is commonly called ophthalmia: it was inadequate to the establishment of a condition in which an unusual quantity of fluid is ejected. The disease in this form is simple at the onset, and it frequently yielded to simple means of remedy in a few days. It rarely assumed a serious form under proper management; and relapses, although not uncommon, were much less so than in the other varieties.

"*Variety II.*—The second variety is of a more complicated nature than the first. The attack is sudden, rarely indicated by premonitory symptoms. Where these take place, they consist of heaviness about the orbits, and inability to move the eye-lids with the usual freedom. The first symptom which attracts the patient's attention, is an increase of the lachrymal secretion, which, as it becomes abundant in quantity, flows over the cheeks, the ducts being incapable of removing it in the usual manner. If the upper lids be examined at this time, although the covering membrane of the ball be free from disease, a villous floculence is there discernible; even sometimes a thickening, approaching to what is commonly called granulation, chiefly observable towards the inner angle, though occasionally over the whole of the eye-lid. It was this state of the eye-lids (and it in many instances precedes the explosion of the disease on the ball of the eye,) which chiefly directed my attention to the plan of treatment hereafter to be noticed. A thickening of the upper no sooner takes place, than the lower eye-lids become villous and slightly prominent. The sensation of sand, if not synchronous with the thickening of the lining membrane of the upper eye-lid, now becomes unpleasant; the patient imagining that the disagreeable feeling he experiences is owing to the lodgment of an extraneous body between the palpebræ and ball of the eye. As the sensation of intervening sand increases, the watery discharge also increases; and, soon becoming acrid and corrosive, produces a degree of pain, which obliges the patient, unaccustomed to the irritation, to seek relief from friction, the act of which produces redness of the sclerotic coat and conjunctival membrane, followed by distension of innumerable small vessels. The meïbomian glands, previously in a state of irritation, now increase in size, and discharge a fluid proportionate to their enlargement. Limpid serum flows from all parts of the eye and eye-lids; and, as it is changed in quality as well as in quantity, in its descent it excoriates the parts over which it passes. Matter of a similar kind insinuates itself into the substance of the lower eye-lids, so as to cause slight tumefaction, and hardness of the parts. From the state of the lower eye-lids, a prognosis may be formed relative to the severity of the disease. The difficulty of cure augments with the tumefaction and hardness of the lower eye-lids; nor can a perfect cure be accomplished until these are restored to their natural flaccid state. In this form of ophthalmia, the action of the secreting vessels is augmented in so high a degree, as not only to cause, in all cases, an extraordinary discharge of fluid, insinuating itself into the surrounding parts, but to produce, in many instances, a defect in vision, by the quantity of serum effused into the anterior and posterior chambers of the eye.

"*Variety III.*—The two preceding conditions of ophthalmia are by many considered as simple. The form now under consideration (viz. the purulent,) comparatively complex. This form of disease, if we permit ourselves to judge by external appearances, is of a more serious nature than the second, but it is doubtful that it is so in reality; for, of the numerous cases of different varieties which came under my observation, those which assumed the purulent mode of action yielded to remedy as soon, and as certainly, as the complicated cases of the other varieties. In this form also the attack is sudden, not announced by premonitory symptoms: when they do exist, they consist, as in the other form, in a sensation of stiffness or heaviness about the orbits,—an inability to move the eye with the accustomed freedom. The eye becomes itchy; and the patient cannot be persuaded but that sand or dirt, as he expresses it, is lodged between the eye-lid and ball of the eye. He endeavours to remove the disagreeable sensation by rubbing; which, not producing the expected relief, is immediately succeeded by obtuse pain, augmentation of the sensation of heaviness, and by a flow of fluid resembling tears; which, from being thin and watery at first, is converted into a puriform matter, wholly divested of the acrimony attending the serous discharge in the second form. If the eye-lids be now examined, a thickening of the lining membrane of the upper is observable, and the lower are villous. As the puriform discharge augments, it becomes dense and tenacious; the capillary vessels on the ball of the eye and neighbouring parts are in a state of irritation. This is considerably augmented by rubbing the ball, and the propensity to rub is irresistible. The vessels which nourish the conjunctiva and lining membranes pour forth a matter of a puriform nature, whilst those of the eye-lids internally, and also of the internal parts of the eye, secrete and discharge a quantity of whey-coloured serum, producing a tumefied state of the eye-lids and of the ball of the eye, which characterize the disease, and occasion the partial or total loss of vision. (The loss of vision is attributable to the existence of a turbid fluid in the aqueous and vitreous humours; the obtuse pain to the distention of the ball of the eye from extravasated fluid, and not to inflammation.) As the turgescence and prominence of the eye-lids, which assume a bluish hue, increase, the vessels which nourish the conjunctiva and sclerotica augment in size; the texture of the former, being reticular and spongy, is injected with red fluid, not blood, or with pure and limpid serum, which, as the determination of fluid to the part is considerable, produces extraordinary distention of the conjunctiva. This is sometimes so great as to cause the distended mass to encroach on the cornea; and, as the size of the latter appears diminished to one-half probably, it excites the apprehension of such as are unacquainted with this form of the disease.

To this state of the conjunctiva oculists have given the name of Chemosis. (Nothing can be more erroneous than that purulent ophthalmia is always connected with, or occasioned by, gonorrhœal discharges. I have seen more than one hundred cases where no venereal taint existed.)" —(P. 5—10.)

We have given our author's description at full length, that we might not do him injustice. His plan of treatment, however, need not detain us long: it is extremely simple, and may be comprised in a very short space; considering these varieties as only modifications of one disease, which, according to our author, is local, the plan of cure was as follows:—The patient was confined to bed in a well-ventilated ward, from which the light was not excluded: he was placed on low diet, purged, and the state of the upper eye-lid especially examined into in every case; and, whenever it appeared villous or thickened, bluestone was rubbed over the surface for a longer or shorter time, according to the state of the membrane. This is at first attended with acute pain, and on one occasion produced syncope; but the resulting benefit is asserted to be very great, and superior to any other hitherto employed. This application is to be followed by fomentations, repeated four or five times in the twenty-four hours. A change for the better will, by these means, be effected in a few hours; and it may be repeated on the second day, if it is demanded; or a solution of lunar caustic, in the proportion of ten grains to an ounce of water, may be instilled into the eye, and the fomentations repeated. Our author speaks warmly of this remedy: he has tried it of all strengths, from one grain to half a drachm to the ounce, but prefers the proportion above mentioned. On the second day it is usual to give a saline purgative, and to repeat the lunar caustic drops in the evening; or to touch the lower eye-lids slightly with the bluestone, and then to foment again. The same plan is pursued on the third day. By this plan, varied according to circumstances, more than eighty in a hundred will recover without the use of other means; and, in consequence of the attention paid to the upper eye-lid, the condition called "granular" will be prevented; relapses will be fewer than under the antiphlogistic mode of treatment; and ulcers will seldom appear, or, when they do, are of a mild kind, and rarely imply much loss of substance.

Such is a brief, but faithful, account of Dr. O'Halloran's treatment of ophthalmia generally; and a reference to Mr. Melin's paper will show how closely the opinions and practice of these gentlemen resemble each other. The latter says that, before he took charge of the ophthalmic division, he considered that a mere local inflammation could scarcely require such extensive depletion as was usually practised; and that he was induced to make trial of a solution of lunar caustic in these cases, from observing its beneficial effects in gonorrhœa, the disease of a membrane similar in structure to the conjunctiva. The strength he employed was four grains to an ounce of water, used twice in the day; since that period he has treated three hundred cases in this way, with complete success; and he produces the testimony of Mr. Beard, of the Artillery, as confirming his views on the subject. But Mr. Melin carefully draws a distinction between cases of superficial and deep-seated inflammation, in which he advocates the early use of the lancet.

Our readers generally, and especially our country readers, will perhaps feel somewhat startled at the doctrines above detailed, and may be inclined to suspect that we are not in earnest with them: nevertheless, we conceive that a little reflection, and attention to a few plain facts, will enable them to reconcile the above practice with the doctrines more usually entertained; and to demonstrate that there does not exist, in truth, so much difference, as they might at first suspect, between the treatment usually pursued in the army and in civil life.

It is not difficult to account for the great prevalence of ophthalmia among soldiers; with them, all the circumstances of their situation combine to produce the disease: they sleep in apartments crowded in comparison to what usually occurs in private life; they are exposed to the heat of the sultry day, to the damp and cold air of night; and are by no means the most temperate or abstemious of mankind. No wonder, then, that, when once ophthalmia is excited, it is kept up for an indefinite period: the same causes are continuing constantly to operate; and, in addition to this, (in a certain state of the disease, at least,) its propagation by contagion can, we think, scarcely be denied or doubted.

We are unwilling to lengthen this article by any discussion upon the subject of contagion. We are aware of the warmth and eagerness with which this particular point was at one time advocated and denied by the opposite factions, if we may so term them; but we shall merely observe, that the contagionist always possesses the vantage ground in these disputes, inasmuch as it is extremely difficult to prove a negative; and because the prejudices of mankind are always operating in favour of that mode of thinking, for which purpose one solitary instance of disease, apparently passing from one individual to another, will, with the majority, always be conclusive. Nevertheless, in the case of ophthalmia, there appears no good reason to doubt that, if the term contagion be restricted to its legitimate meaning, the matter secreted from the conjunctiva, or probably the tears themselves, are capable of generating a similar disease,

We have, for our own parts, so often seen what appears to us to be irrefragable proofs of this inoculation (if it may be so called), that there is no fact in medicine or surgery more strongly impressed upon our minds. Ophthalmia, then, is a disease with which the army surgeon is unfortunately familiar: in one respect, however, he may be considered as fortunate, because he has the opportunity of seeing the disease absolutely in its nascent state. From him there can be no concealment,—no waiting for a day or two, in the hope of becoming better; but he is at once enabled to adopt those measures which appear to him to be requisite.

Upon this simple circumstance we are induced to found the success of the methods recommended by Dr. O'Halloran and Mr. Melin. The latter of these gentlemen says that he adopted the practice he recommends, from observing a similar good effect to ensue from the use of the same remedy (the lunar caustic) in gonorrhœa. This is another proof in confirmation of our opinion; for every surgeon knows that counter-irritants, applied in the very commencement of a gonorrhœa, will frequently, nay generally, arrest the complaint; and yet these very remedies, if employed after the inflammatory symptoms have fully developed themselves, would produce, and have produced, wherever they have been either inadvertently or injudiciously recommended, the most serious consequences. Again, Dr. O'Halloran says, ophthalmia is a local disease; another proof that he is speaking of it in its very infancy: for surely no one will venture to say this of an ophthalmia which has proceeded in its course a given time, and where we shall find the whole arterial system in arms, and the constitution generally sympathising in the attack. This opinion is still more strengthened by that gentleman himself, when he says that this state of the eye-lids (the thickened), in many instances *precedes* the explosion of the disease on the ball of the eye; that being, in truth, the period when the lunar caustic, or sulphate of copper, may be used with the view of cutting short the disease.

On another point we must likewise offer a few words. Dr. O'Halloran exposes his patients freely to air and to light. With regard to the former of these directions, we agree with him perfectly: we consider the exclusion of air, by close shades or clothes, as highly pernicious; but we cannot express the same opinion as far as light is concerned, since nothing can be more intolerable or painful to patients labouring under any condition of inflammatory action about the eye: nay, we will venture to say that the eye will instantly shut itself against the light, whatever the wish or the command of the surgeon may be. It appears to us, therefore, that, although we must admit that Dr. O'Halloran has made out a good case in favour of his mode of treatment, that those who practise in civil life will do well to recollect that it is only adapted to the *very commencement* of the disease; that where local pain is great,—where the sclerotic coat, or any of the internal parts of the eye, partake of the inflammatory action,—where the constitution feels the disturbance,—depletion, rational but effective depletion, must still be our sheet-anchor. We may confess freely, that this practice has been carried too far, though we certainly have never seen it pursued to the risk and hazard of life, as asserted by our author; but still we do maintain, that we are by no means so convinced by what we have read, as to recommend the unrestricted adoption of the measures above mentioned, which would seem to us to be little less than overturning all the established principles of surgery, by which we are taught that acute inflammation is best combated by blood-letting; and, to say in the words of a highly-esteemed writer on this disease, that experience has demonstrated to us, over and over again, "that the relief afforded by venesection in this class of complaints, is *indescribable*." We, therefore, are of opinion that our author has not sufficiently explained the principles upon which he acts; that he has not defined accurately those conditions of disease in which this stimulating plan is to be adopted; that he has not sufficiently warned us of the necessity of restricting its application to the affections of the conjunctiva simply; a fault which Mr. Melin's papers are entirely free from.

Dr. O'Halloran's chapter on the *acute ophthalmia* is only illustrated by one case.

We now proceed to examine the second chapter, containing practical remarks on *chronic ophthalmia*, from which our extracts will be but sparing, as there is but little difference of opinion among surgeons as to the treatment of this form of the disease: indeed, the principal motive for dwelling upon this chapter at all, is to lay before our readers two peculiar opinions broached by our author; one of which is theoretical, the other appears to us to be merely a dispute about a word. The first opinion we shall give in the Doctor's own words:—

"I am of opinion that, in all cases of ophthalmia of the acute kind, the action of the arteries is inordinately increased, while that of the veins is stationary or diminished; and that upon the relative degrees of action in both depends the nature of the discharge. Where the action of the arteries is moderately excited, with or without a corresponding change in the action of the veins, a watery discharge is produced; where the action of the arteries is inordinately roused, with a corresponding defect in the action of the veins, a purulent production is the consequence:—this appears to be the case in all forms of the disease. The determination of fluid to the parts is inordinate; and, as there appears to be a defect in the activity of the absorbents, or organs of removal, the action of the glands being also augmented, through the irritation occasioned by immoderate distention, an effusion takes place, both vascular and glandular.

"In chronic ophthalmia, on the other hand, although a state of activity exists in the trunks of the arteries, the blood is torpid in the minute branches; whilst a near approach to stagnation takes place in the veins, external as well as internal. In this case, as well as in the former (viz. the acute), the discharge of fluid from the eye is considerable; for the glands, whose action is augmented according to the increase of disease and size, emit a fluid in quantity considerably exceeding, perhaps, that which is poured out in the acute stage. If we apply caustic or bluestone to the upper or under eye-lids in the chronic ophthalmia, so as to produce a slough, the vessels, being destitute of power, are unable to throw it off in less than from three to six days; whilst, in the acute stage, where the arteries are possessed of high action, the same quantity of slough is ordinarily removed in one night. This is a fact of practical importance. It furnishes us with the means of ascertaining whether the case before us be of the acute or chronic character; and no injury can result from the experiment, for bluestone is equally serviceable in both stages: in addition to this, we also find that, when the disease assumes the chronic form, the veins on the upper eye-lids, forehead, and temples, are gorged with blue blood, and, as they appear wholly destitute of activity, the blood which they contain is in a state of demi-coagulation; the arteries also of the ball of the eye are inordinately distended, and hard to the touch; they appear destitute of the powers of propulsion. I have given this subject a considerable share of attention; and I am of opinion, that a diagnostic can only be formed from the effects of remedies on the parts, or from the state of the vessels external and internal."—(P. 25—27.)

Notwithstanding Dr. O'Halloran's assertion, that he has given this subject much attention, we cannot help thinking that, to form a diagnosis from the mere effect of remedies, is but an uncertain and dangerous mode of proceeding, because, if the practitioner finds himself in the wrong, he cannot well undo the mischief he has done; and we do not see any reason for disregarding the old-fashioned symptom of *acute pain*, as a much surer guide in forming our judgment of the nature of the disease.

With regard to the second point to which we have alluded above, it refers to the existence of granulations in the eye-lids, a circumstance which Dr. O'Halloran utterly denies; and he is equally severe upon the employment of the scissars or knife to remove them. We think well of this practical rule, and believe it to be perfectly just; but, when he denies the existence of granulations, we conceive he is combating with a shadow:—he himself uses the term "granular" in the sense in which it is commonly employed. He recommends caustic or bluestone for the purpose of getting rid of this diseased condition of the membrane; and his whole quarrel seems to be with the word granulation, to which he has attached the same meaning as is usually applied to the same appearances in open ulcers; a point for which no surgeon, we apprehend, would contest.

We have seen above that our author condemns the use of the knife or scissars; yet there is one condition where the knife, he says, is admissible: it is in the case of the eye-lids being covered with fatty, dry lumps.

The last eight or nine pages of this chapter are occupied with criticisms on detached portions of Mr. TRAVERS' work; the tenor of which is to prove that oculists are, upon the whole, undecided with regard to the treatment of ophthalmia. Having already spoken our sentiments as to the real merits of Mr. Travers' work, we have no intention of becoming the critic of these criticisms; but we think that Dr. O'Halloran is wrong, if he means to employ the term "oculist" to designate that able and intelligent surgeon; and we moreover think that, as a whole, Mr. Travers' book will be found to contain as sound directions for the treatment of diseases of the eyes as any work in our language; and that it is hardly fair to select detached passages of a book for comment, which, taken in connexion with what precedes and follows them, may admit of an interpretation altogether different.

We are happy, in concluding our remarks on Dr. O'Halloran's publication, to be enabled to say that the third chapter, on *ulcers of the cornea and lymphous effusion*, is replete with good sense, and with valuable practical remarks, detailed in a very perspicuous manner; and that, if we are content with this general praise, and do not lay before our readers a formal analysis of this chapter, it is partly because this article has extended so much further than we at first anticipated, but more especially because the doctrines contained in it do not, in any material point, differ from those entertained by other enlightened practitioners.

Mr. HEWSON's Treatise on that form of Ophthalmia accompanying the secondary stage of Lues Venerea, next claims our notice; and we feel great pleasure in being able to say, that the task which this gentleman has imposed upon himself has been executed by him in a very creditable manner. Our author complains, and with reason, that this symptom of syphilis has not been attended to sufficiently by systematic writers, nor described with any thing like accuracy even by those who have professedly treated of the disease itself; and he instances some of the greatest names in surgery, in proof of his assertion. From this reproach the late Mr. SAUNDERS is, however, very properly excepted; and it is only to be lamented that the untimely death of that promising surgeon "left this, as well as most of his other labours, unfinished." Such are the principal considerations that have induced Mr. Hewson to think that a short and distinct practical view of the venereal ophthalmia, divested of those *speculative doctrines* in which the whole subject of lues venerea has, within these few years, been involved, was yet wanting; and he very modestly adds, "that, though an experienced reader will find nothing new or interesting in it, I trust its perusal will prove of some advantage to those for whose instruction it was chiefly undertaken, and is now offered to the public."

We have printed in italics the only part of this paragraph with which we intend to quarrel: we think that Mr. Hewson is not justified in designating the late inquiries into the natural history of lues venerea, as speculative doctrines. Nothing less speculative was ever offered to the public; since from experiment only have all the deductions been drawn, and these experiments have not only been made upon the most extensive scale, but appear to us to have been conducted in the fairest and most ingenuous manner.

In describing this form of ophthalmia, our author observes, that it is useful to distinguish the symptoms into two stages: in the first, they are effectually curable; in the second, they are in a great degree out of the reach of relief, the structure of the eye being more or less permanently injured. This disease always first attacks one eye, though in long-neglected cases this does not invariably happen, and sometimes they suffer alternately. The first attack of the complaint is not attended with any great pain; there is an increased flow of tears, and objects appear as if seen through a mist. We copy the following accurate description of the symptoms, as given by our author:—

"The patient keeps his eye half-closed, and avoids, as much as possible, exposure to the light, which always excites pain and flow of tears. From the same cause the eye rolls, and is so unsteady, whilst an examination is making of it, that it is often difficult to get a satisfactory view of it.

"There appears always more or less external inflammation; and if we attentively examine the enlarged vessels, we shall, in general, be able to distinguish two tissues of them; one superficial, which belongs to the conjunctiva; and the other more deep-seated, connected with the substance of the sclerotic: in colour, they are sometimes of a florid, but oftener of a purple or venous hue. That part of the conjunctiva which lines the eye-lids is not usually under much irritation; the portion which covers the eye-ball is a little more inflamed: sometimes it is here a little œdematous, or a small vesicle or two may be observed on it; but it never enters into the thickened, fungous structure, nor is there any secretion of purulent mucus from its surface; both of which are so often the effects of the severer forms of external ophthalmia.

"A great part, however, of the redness of the eye-ball is produced by vessels passing through the sclerotic, which, becoming more numerous and frequent in their inosculations as they advance forward, and terminating abruptly, nearly at the space of a line from the cornea, about the situation of the ciliary ligament, cause in this part the peculiar appearance of almost a distinct red circle. Neither at this or the more advanced periods of the disease, do these enlarged vessels pass over the surface of the cornea; nor have I ever observed any speck or ulcer forming on it: in a few instances there has been an appearance like the arcus senilis." (P. 4, 5.)

An inexperienced observer may be at a loss to account for the great defect of vision, of which the patient complains; but the state of the aqueous humor and pupil will account for this: an opaque fluid is seen floating in the anterior chamber, preventing a clear view of the iris and pupil. This is variable in quantity; and, in advanced stages of the disease, vessels shoot into it, and it becomes organized: occasionally, instead of lymph, blood is effused. The state of the pupil is various: as the disease advances, the pupil contracts, and loses its circular shape; but the superior part more usually preserves its natural form. In many cases, one or more tubercles present themselves about it; but they more properly belong to the second stage. The colour of the iris undergoes a very remarkable change: sometimes its surface is found dotted with spots; but our author does not lay much stress on this appearance. Such is the general description of these symptoms, which may remain from six to eighteen days, being seldom protracted beyond the twentieth; and at this period, when the pupil is contracted, and its motions impeded, adhesions may form between it and the capsule of the lens, without being immediately perceptible.

We must hurry over the symptoms of the second stage; but we are told that the choroid coat and retina partake of the diseased action, as well as the iris, and that about this period the other eye begins to be attacked; at first, the symptoms of the complaint appear to be suspended in the eye first attacked, and are afterwards renewed; in this way the eyes are alternately affected, until the disease has fully run its course in both. In some instances the symptoms will afterwards assume a more indolent form, and the pain and irritation will gradually and finally subside. The most fatal termination of the disease is that wherein an abscess forms in the interior of the eye; the first symptom indicating which is some degree of œdema on the fore part and one side of the eye-ball, immediately behind the ciliary attachment of the iris; but such cases are not common. With respect to prognosis,—it is in general favourable, unless when the mild and insidious nature of the attack induces the patient to delay seeking relief.

Mr. Hewson next adverts to the constitutional symptoms of lues, which are commonly found in conjunction with this form of ophthalmia: he is of opinion that this will generally be most severe where the constitutional symptoms produce most disturbance; and that it assumes a more exasperated form where no mercury has been given for the cure of the primary symptoms.

Venereal ophthalmia is commonly accompanied by some form of eruption, or affection of the throat: the eruptions most usually attending it are the scaly and papular; but he believes that it may be associated with any general symptom, or exist independent of any. This latter circumstance we are inclined to think very rare, and believe that most cases, closely examined into, would exhibit some other form of disease in conjunction with it.

The general appearance of a patient labouring under these various symptoms is so striking, that we need only say that it is well described by our author. Among women, it is to be recollected that sometimes the only circumstance connecting this symptom with syphilis, is their having had repeated abortions, or still-born children.

On the subject of exciting causes, we find nothing to extract; and we therefore proceed to enumerate some affections of the eye, with which our author conceives this disease may be confounded. The first of these is idiopathic inflammation of the iris: and here we must confess that our author's diagnostic signs appear to be of little practical utility; for, to judge of the different nature of a disease by its different termination, would be but poor satisfaction to the sufferer. In fact, Mr. Hewson himself confesses that the state of the patient's general health, and the history of the case, must be investigated. It appears that, in Ireland, the necessity of making this inquiry has been peculiarly strong, from the circumstance of iritis having frequently occurred as a sequela of typhous fever. Secondly, from inflammation of the capsule of the aqueous humor, the venereal ophthalmia has been discriminated by Mr. Wardrop; and we do not find our author adds any thing material to what that gentleman has pointed out: the same remark applies to rheumatic ophthalmia. Our author doubts the existence of arthritic ophthalmia: at least, there can be but little fear of mistaking this for the disease now under consideration.

In considering the ophthalmic symptoms caused by mercury, we find Mr. Hewson controverting the opinion of Mr. Travers. He appears to think that it is the inefficient, or merely alterative, exhibition of mercury, when the patient is placed under no restraint, that causes the frequent recurrence of this, as well as many other constitutional symptoms of the disease; and that iritis is by no means a frequent result of the constitutional use of mercury: on the contrary, he says, that these symptoms are of a very distinct and different nature; the external structures of the eye being more commonly affected by it, and the conjunctival ophthalmia, especially, being prolonged and exasperated by its use. We shall scarcely do our author justice upon this much-contested point, without giving in his own words the concluding remarks on this subject:—

"The effects which I have above ascribed to the morbid agency of mercury, are of a nature peculiarly liable to escape observation; and probably from thence have not been at all attended to. I would not be understood as maintaining that they are often the consequence of a short and temporary use of mercury, though in some instances this too was observed; but I have found them so repeatedly resulting from a negligent and protracted exhibition of it, particularly where it was not calculated to act as a remedy, that I could not avoid taking this opportunity of fully stating my views on the subject; more particularly as it is much inculcated, and generally believed, that the constitutional use of mercury may be resorted to, not alone with impunity, but advantage, in all species and states of ophthalmia. If, however, the observations that I have made are well founded, this must appear to be a practice productive of very extensive mischief, and should be guardedly refrained from.

"From what I have also stated above, the differences between the ophthalmic symptoms directly and exclusively dependent on the constitutional use of mercury, and those we have seen attending syphilis, will be sufficiently evident. However, in those complicated and anomalous cases, which are but imperfectly known to us under the title of pseudo-syphilis and other denominations, and in which I believe there are grounds for suspecting that the syphilitic and mercurial poisons are often operating on the system at the same time, we shall frequently find syphilitic symptoms, or symptoms precisely similar to them, attacking the eyes as they do other parts. It is here extremely difficult, if not impossible, to form any diagnosis; but, at the same time, this is not necessary; for we must always treat ophthalmic symptoms occurring under these circumstances, and of this nature, as if they were of a genuine and unmixed syphilitic origin.

"It is, therefore, to be observed, that the plan generally adopted, and which experience fully sanctions, of withholding mercury in most cases coming under the description of pseudo-syphilis, is to be deviated from when any symptoms, however remotely, characteristic of a venereal nature, attack the eyes: for, if we here wait the slow effects of any other treatment than that with mercury, these delicate organs will, with certainty, sustain irreparable injury." (P. 53—55.)

We come now to the mode of treatment recommended by our author, which will not detain us long. It fortunately happens that a judicious use of mercury overcomes this symptom, as speedily and as certainly as any that are found to accompany the lues venerea. Mr. Hewson reprobates the employment of the corrosive sublimate, as practised and recommended by many continental surgeons, and we join him in this opinion; yet we think sometimes, when we have been anxious quickly to produce the effects of mercury, we have found it advantageous; but we would not rely upon it as a mode of cure. Neither bleeding nor blistering are of any avail in this disease. Stimulating collyria also aggravate the sufferings of the patient; and, if any local application be found necessary, the poppy fomentation will generally be found to be the most grateful. Mercury, therefore, is the grand remedy, and confinement can less be dispensed with than in that of any other symptom of lues. We are to recollect that, when the morbid action is removed, and the functions of the eye are restored, the exhibition of mercury must not be stopped, but this remedy must be persevered in until the constitution is cleared of the poison.

Our author concludes this portion of his work with some remarks upon Mr. Travers' opinion, that all forms of iritis require the constitutional use of mercury for their cure, without exception. Mr. Hewson thinks, on the contrary, that many cases of iritis depend upon disorders of the digestive functions, or upon morbid excitement or action of the brain, and is therefore anxious to restrict the use of mercury in this form of disease to the most precise limits. We confess that we are not convinced by what our author urges upon this point. That there may be such cases as he mentions, we do not deny; but we still think that, in the treatment of iritis, from whatever cause, mercury will be found to be the sheet-anchor.

We regret that we cannot insert any of the very interesting cases that close this volume; but we regret it the less, because we have said enough, we hope, to induce our readers to turn to the original work; from the perusal of which, our short and imperfect analysis cannot, and ought not, to exonerate them.

---

Art. IV.—*Observations on Fever.* By R. Wade, Member of the Royal College of Surgeons, and Apothecary to the Westminster General Dispensary.—8vo. pp. 83. Burgess and Hill, London, 1824.

This little volume comes before us in a very unassuming manner, and, with little pretension, contains more practical information than many, the title-pages of which constitute a little essay, setting forth all that the book does contain—and much which it does not. If, therefore, the work which we have placed at the head of this article be no rival to the classical volumes of Bateman and Armstrong, so neither does it provoke the comparison; being both intended and fitted for the perusal of students, who either have not the opportunity of reading,—or, reading, fail "inwardly to digest" the stronger intellectual repast which the writings of these authors contain. It is with a view of drawing the attention of the junior members of the profession to an Essay designed and calculated for their advantage, that we give it a place thus early in the commencement of a new season of study—and it is to be hoped of improvement. The motives which induced the author to become a candidate for public attention, as well as his general views regarding the treatment of fever, will appear from the following quotation:—

"In offering the following observations to the notice of students, for whose use they are expressly intended, I must beg to explain my reasons for thus occupying their attention. Having been constantly with pupils during the last five years, I have had various opportunities of ascertaining their opinions of disease. When questioning them as to their views regarding the theory and practice in fever, scarcely any of them have been at all decided in the course they should adopt, not knowing which of the many theories, and consequent modes of treatment, to prefer. Blood-letting has great charms with most students; there is at once a boldness and decision in the remedy, which pleases them; besides, it is surgical. It has appeared to me, of late, that their great error has been to treat fever more like a mere local inflammation, than as a disease which is probably to last for two or three weeks, and perhaps longer. Even the works of Clutterbuck and Armstrong, which have so much improved the pathology of fever, (and which cannot be spoken of too highly,) are quoted by pupils as a sanction to their most daring achievements with the lancet. We are all too apt to run into extremes, instead of prudently adopting the maxim of the poet—"Medio tutissimus ibis." To cause the student to reflect well as to what extent he may safely carry bleeding in continued fever,—to call particularly his attention to all those remedies which will frequently prevent the loss of blood,—and more especially to impress on his mind the wide difference between the treatment of a simple local inflammation and continued fever, has been a great object in the ensuing remarks." (*Preface*, p. v. vi.)

After some preliminary remarks, written with considerable force, to enjoin industry and perseverance in study, with the remark, that the "plodding man frequently grows fat, whilst the man of genius starves," Mr. Wade proceeds to give a rapid sketch of some of the systems which formerly prevailed in the schools; in the course of which, he takes occasion to inveigh against the humoral pathology, as an "unintelligible jargon," and leading to "injurious practice." That this doctrine was rendered unintelligible by the complicated refinements of its cultivators, and even led to injurious practice by the hypothetical views of disease which it consequently gave rise to, we acknowledge: yet we are free to confess that, in our opinion, there are many principles of the humoral pathology, which, however unfashionable, are nevertheless founded in truth, and capable of useful practical application.

The disadvantages resulting from limited views of disease, and from the exclusive devotion to the fashionable theory of the day, or the authority of favourite teachers, are forcibly, but not too strongly, described in these words:—

"The man who regards a disease with any particular theory, may be compared to one who surveys different objects through a coloured glass, when, however various their hues may in reality be, still each will receive a peculiar tint from the medium through which they are surveyed. I do not mean to say that it is impossible to establish any general principles in fever, but merely that they should be received with great caution, taking care to make them subservient to facts, and not, as is too frequently the case, making facts give way to theories.

"Considering, then, the difference of fevers, and the variety of organs that may suffer, surely it is a most dangerous thing here to generalize much; for, although to the man of experience it may do little harm, as he will rather be guided by his practice than theory, yet on the young practitioner it will produce a very different effect; for early impressions are not easily effaced, and he will besides naturally feel a wish to support the doctrines of his teacher.

"Every young man, when first entering on the practical duties of his profession, and encountering its fearful responsibilities, must have met with many a bitter disappointment in practice, finding in every day's experience too many of our boasted remedies falling far short in virtue to what his reading had taught him to expect. He has read the best descriptions of, and attended the best lectures on diseases; their remedies, perhaps, are as familiar to him as their symptoms. He knows the structure of the human body,—the accidental injuries to which it is subject; can describe with accuracy the different operations which may be performed for their cure. With all this knowledge, however, he will be like one who has read the best descriptions of a country, the customs and manners of its inhabitants; yet, never having himself travelled through the parts of which he has read, should occasion call upon him to visit them, he will not only find many things to learn, but that he has also formed many erroneous conclusions, which a minute survey of the country, and a continued observation on the character of its inhabitants, can alone enable him to correct.

"Different writers, and those men of the greatest genius, have given us laws by which to regulate our practice; yet their very genius has frequently led them astray, and, by entering into wide speculations, they have done more harm by influencing the practice of their less gifted brethren, than good by their many valuable observations. The science of medicine has seldom been much advanced by imagination, and the plain narrator of facts will do more real good than the framer of systems. In youth, when fancy reigns uncontrolled by experience, and caution has little sway, theories are most dangerous; for the mind embraces them with eagerness, and, as it were, at one grasp arrives at conclusions which years of toil would not, perhaps, enable it to obtain.

"The student is, indeed, placed in an awkward situation as regards his practice in fever, so very opposite are the treatments recommended by different writers: one tells him to bleed largely at the commencement of typhus, and that, as he diminishes the excitement in its early stage, so will he prevent the ensuing debility;—another will tell him that, should he bleed in typhus, he will kill nineteen patients out of twenty: this last writer, no such friend of Sangrado as the preceding one, quite damps his ardour for the use of the lancet. Perhaps a third, being very fond himself of using the cold affusion, will highly extol this remedy, and advise him to rely chiefly upon it; pursuing, at the same time, the antiphlogistic plan strictly. From reading the successful result of the practice of this last author, ascribed almost entirely to the cold affusion, and being more than ever impressed with the virtues of cold water, (its simplicity also recommending it,) he will probably determine to adopt this practice. It may so happen that the very next author he reads will quite freeze all this cold-water mania, and frighten him out of its use, by saying, if he ever have recourse to the cold affusion, which it is not often safe to do, that it requires great caution in its management. He will also tell him that many patients have never recovered from the shock occasioned by it, not having sufficient powers for the necessary reaction; or, if there be sufficient power, that it frequently does great harm, by occasioning a determination of blood to some internal part, whose vessels are in a weak state, and thus gives rise to local disease. One would naturally suppose that all difficulty on the part of the student would be got over by comparing the different methods of treatment, and of course adopting that which was most efficacious; yet, however extraordinary, each writer, for the most part, brings forward such strong evidence in favour of his particular plan of treatment, that the reader is as much in the dark as ever as to which he must pursue, and is, in fact, left to the sage conclusion that all are equally good." (P. 10—14.)

To lay the views of the author before our readers in succession, could only be done by compressing still more that which is sufficiently concise already; we, therefore, pass over some brief, but judicious, remarks upon contagion and the effect of charms,—upon the brain and nerves, as connected with fever,—upon the morbid appearances found on dissection,—upon the difference of fevers,—their usual symptoms, particularly delirium and coma,—upon the prognosis,—and, lastly, the treatment. After general remarks upon this last subject, the author proceeds to their illustration, by giving an imaginary case.

"Suppose we are called to a patient in a respectable situation in life, accustomed to good living, and enjoying generally a good state of health;—let us further suppose that there is no reason to regard his fever as epidemic, or as having arisen from contagion;—he complains of considerable pain in the head, his countenance is flushed and anxious, pulse hard and quick, besides the symptoms usually attending fever.

In this case no doubt can be entertained as to the propriety of general bleeding, which should immediately be had recourse to, until the patient complains of feeling faint, or the arterial action be considerably reduced. The next object in view is to remove the contents of the stomach and bowels, knowing that the different secretions acting as solvents of the food, and assisting in its digestion, are now interrupted, or suppressed; and, consequently, that the contents of the alimentary canal must act as a source of additional irritation to that which already exists. An emetic should, therefore, be given; and, if the arterial action be strong, one which tolerably reduces the powers of the system ought to be preferred:—perhaps, to an adult, either of the following will be as good as any for this purpose:

℞. Antim. Tart. gr. ij.
Sacch. alb. ℈j. M.

or, ℞. Antim. Tartar. gr. j.
Pulv. Ipecac. gr. xv. M.

They should be given in a wine-glass full of warm water; and, when beginning to operate, the patient should also be desired to drink freely of warm water.

" As soon as the stomach becomes quiet, a purgative should be administered.

1. ℞. Hydrarg. Submur. gr. vj.

Pulv. Jalapæ, gr. x.

Pulv. Zinjib. gr. ij. M.

2. ℞. Hydrarg. Submur. gr. iv.
Pulv. Rhei, gr. viij.
—— Zinjib. gr. ij. M.

3. ℞. Pulv. Antimon. P. L. gr. v.
Hydrarg. Submur. gr. iv. M." (P. 49, 50.)

We prefer the second emetic to the first, as, according to our experience, free vomiting is more likely to be excited by the addition of ipecacuanha, than by the larger dose of tartarized antimony;—and the third purgative would have pleased us better, had the pulvis Jacobi (veri) stood in the place of the pulvis antimonialis. Nothing appears to us a greater mistake than the general opinion, that these two antimonial preparations are similar, and capable of being advantageously used as substitutes for each other.—But to proceed.

" We must now endeavour to determine to the skin, and keep up a gentle action of the bowels; in short, to keep down the arterial action as much as circumstances may require: either of the following mixtures will answer our purpose:—

1. ℞. Potassæ Nitrat. ʒj.

Træ. Digit. fʒj.

Vin. Colchici fʒij.

Aq. distillat. q. s. f℥viij. M.

Sumat cochl. iij. magna tertia vel quarta quaque hora.

2. ℞. Sulphat. Magnesiæ ℥j.
Antim. Tart. gr. ij.
Aq. distillat. f℥viij. M.
Sumat cochl. iij. magna tertia vel quarta quaque hora.

3. ℞. Magnes. Sulphat. ℥ss.

Potassæ Carbon. ʒjss.

Succi Limon. q. s. (ad saturandum)

Vini Antim. Tart. fʒj.

Aq. distillat. q. s. f℥viij. M.

Sumat cochl. iij. magna tertia quaquæ hora.

"If the symptoms of excitement run high, the second mixture will be the best; if not, either of the others: the quantity of magnes. sulphat. or antim. tart. will, of course, be varied as the state of the bowels or nausea may indicate. A little syrup may be added, should the practitioner think it an improvement: he will not, however, be able to make them very palatable; but let him be guided by his taste." (P. 51, 52.)

Of these mixtures we greatly prefer the second as a prescription of general utility, having but little faith in the power of digitalis or colchicum, under such circumstances. If the pain continue at our next visit, and the pulse be still quick and hard, we are advised to draw more blood from the arm, and continue the same medicines; and if, on the following day, the pain in the head and excitement continue, leeches are to be applied to the temples. In addition to which means, the cold affusion may be had recourse to, if the heat be steadily above the natural standard, and there be no local determination. We cannot follow this patient further, but proceed to contrast him with the second example.

"Let us, for another example, imagine the same patient having the symptoms just described; but, instead of his fever arising from cold, fatigue, or other causes, we have every reason to conclude that it is epidemic, or proceeding from contagion,—what difference would this make in the treatment? If called as early as in the first case, we may commence with the same measures,—that is, the bleeding, followed by an emetic and cathartic; but the next day, instead of general bleeding, it will be better to rely upon cupping or leeching, unless active inflammation be going on in the brain, or other organ of considerable importance, which may force us to adopt both general and local bleeding. One of the mixtures ordered in the first case should be prescribed; and here those remedies which assist in reducing arterial action are very valuable. Small doses of calomel, with James's powder, or the pulvis antimonialis P. L., may be given at bed-time. The same rules as before must guide us in using the cold affusion: in short, every means which can prevent the necessity of employing the lancet should be had recourse to, we must be as well prepared as possible to meet a typhoid stage; knowing that fevers arising from contagion have a tendency to assume a low character. In this case, suppose that the treatment adopted has appeared to reduce the general excitement; but, instead of the pulse becoming softer and less frequent, it is equally quick, although diminished in force, and of a wiry or thrilling character; that the secretions, instead of returning to a more healthy state, become more vitiated, the tongue is now covered with a brown fur in its centre, and perhaps red around its edges; the general restlessness is changed to a listless state; the feculent evacuations are fetid and slimy; the mental actions, instead of being more excited, are now more languid: here then we have typhoid symptoms, and, when once these are established, I much question whether general bleeding be admissible, although inflammation may appear almost imperatively to demand it, and even local bleeding must be used sparingly; but it is, perhaps, one of the most difficult points in practice to decide as to the propriety of bleeding when a patient is threatened with extreme debility on the one hand, and on the other with severe local inflammation; it is one that great experience and observation can alone decide. In this stage either of the following mixtures may be given, varied according to circumstances;—

℞. Acid. muriat. fʒss.

Aq. distillat. f℥viij. M.

Sumat cochl. duo magna 2dis. vel 3tiis. horis.

℞. Sp. Æther comp. fʒij.

Liq. Ammon. acet.

Mist. Camph. āā f℥ij.

Aq. distillat. q. s. f℥viij. M.

Sumat cochlearia tria magna tertia quaque hora:

℞. Potassæ Carbon. ʒjss.

Succi Limon. q. s. (ad saturandum)

Sp. Æther Nit. fʒij.

Aquæ distillat. q. s. f℥viij. M.

Capiat cochl. iij. magna tertia quaque hora." (P. 55—57.)

These mixtures are well adapted to the circumstances described; but, in general, the decoct. hordei will be found a more eligible vehicle for the muriatic acid than the plain distilled water. Mr. Wade does not conclude the treatment here, but goes on to inform us, that if, notwithstanding these remedies, the patient falls into that state known by the expression collapse, we must have recourse to the free administration of stimulants, such as wine, brandy, ether, tincture of opium, &c.

A third case is put, into the particulars of which we cannot enter; it is descriptive of fever as it occurs among the poorer classes of society in large cities. Some observations follow on particular remedies; and we agree with all that is said of emetics, cathartics, calomel, blisters, opium, and wine: respecting colchicum and digitalis, as remedies in our common continued fevers, we have already expressed our opinion.

The work concludes with general directions for the management of the patient, and, what is sometimes more difficult—for the management of the nurse. These are good, and contain many little points of real importance, although too little attended to in general.

In conclusion, we beg to state our conviction, that the remarks of Mr. Wade have been deduced from accurate observation at the bedside of the patient, and consequently are likely to assist those who would study disease as it occurs in nature, and who do not confine their knowledge to the descriptions of it given in their books.

---

### DIVISION II.

### FOREIGN.

ART. V.—*Recherches nouvelles et Observations pratiques sur le Croup, et sur la Coqueluche, suivies de Considerations sur plusieurs Maladies de la Poitrine et du Conduit de la Respiration, dans l'Enfance* et *dans la Jeunesse.* Par THEODORE GUIBERT, Docteur in Médecine de la Faculté de Paris, &c. &c. &c.—8vo. pp. 326. Paris: chez Béchet. 1824.

*New Researches and practical Observations on the Croup and Hooping Cough, followed by Observations on numerous Diseases of the Chest and Windpipe, in Infancy and Youth.* By THEODORE GUIBERT, &c. &c.

OF all the diseases to which children are obnoxious, there are few, if any, which more imperatively demand the utmost attention of the practitioner, than those which are treated of in the present volume. Notwithstanding the various works which have been presented to the profession upon these complaints, there still exists much discrepancy of opinion as to the best mode of treating them, and even their very nature is not unfrequently a subject of dispute. It is not the intention of Dr. GUIBERT to give a complete treatise upon these affections: he proposes merely to state the result of his practical experience; and, from having been connected with an extensive establishment which is dedicated to the diseases of children, his opportunities have been necessarily greater than the private practitioner can generally enjoy. We have now to examine the use he has made of these opportunities, and to present to our readers a condensed view of the volume before us. It is divided into three parts.

The first part treats exclusively of Croup. The various denominations which have been applied by different writers to signify this disease, are first enumerated, and we then enter upon the practical observations of our author. Croup he considers to be almost exclusively a disease of infancy. It is characterized by the acuteness of its progress, and the invariable tendency to form a false membrane in the interior of the trachea. The seat of croup is stated to be in the mucous membrane which lines the respiratory canal, from the commencement of the larynx to the first division of the bronchiæ. The inflammation may invade the whole extent of this membrane, or only attack a part of it, as has been demonstrated by post-mortem examinations. That the inflammation of croup is exclusively confined to the trachea and bronchiæ, is the general opinion. The investigations of Dr. CHEYNE* and Dr. BAILLIE,† however, have proved that it not unfrequently extends to the lungs, and produces extensive and irremediable mischief. Dr. Guibert considers the nature of croup to be essentially and primitively inflammatory, and does not think it possible to admit a nervous species of croup, which is unattended by inflammation; for, he observes, "the very nature of the symptoms,—the fever, the local pain, the swelling of the neck, the redness of the mucous membrane lining the air-passages, which dissection has constantly detected, and the production of the adventitious membrane in the trachea, all indicate an inflammatory action from the very commencement to the termination of the disease." This we believe to be a very correct view of the nature of croup. That it is always essentially an inflammatory disease, we have no doubt, notwithstanding the frequency with which we hear, in practice, of cases of "spasmodic croup." But, although such is our opinion of the essential characteristic of croup, we believe that many of the fatal cases which occur, in the second stage of the disease particularly, at a moment when we have every reason to believe that the severity of the disease has been checked, must be attributed to spasm of the muscles of the glottis, rather than to the intensity of inflammatory action. We are aware that this opinion has been strongly opposed by very respectable authority; but we are unable to account for the occasional occurrence of *sudden* death in cases of croup, which we more than once have had reason to lament in our own practice, when we flattered ourselves, from the subsidence of the inflammatory symptoms, that we had conquered the disease.

In common with most other authorities, M. Guibert is of opinion that croup is never a contagious disease. It frequently exists as an epidemic; and in cold and humid countries, which are exposed to sudden vicissitudes of temperature, it is sometimes endemic.

There are many anatomical and physiological circumstances, which tend to render croup so much more frequent in children than in adults, and to give to the disease its pathognomonic symptoms. Such are, particularly, the imperfect development

* *Pathology of the Membrane of the Larynx.*
† *Morbid Anatomy.*

of the air-passages, and of the glottis especially; the great sensibility of the mucous system in general, in which the membrane of the respiratory canal participates; the extreme difficulty of expectoration in children, which favours the accumulation of the mucous secretion, and gives rise to the obstruction of the bronchiæ; the activity of the circulation, which explains the easy development of inflammatory affections at an early age, as well as the severity of the symptoms, and the rapidity of their progress. "Croup attacks indifferently children of both sexes, and almost as frequently the feeble as those who enjoy a robust constitution."

Among other exciting causes of the complaint, which our author enumerates, he mentions the introduction of a foreign body into the larynx or trachea. That genuine croup should ever arise from this cause, is very improbable. Much distress will necessarily be occasioned by the presence of any foreign body in so irritable a canal. Inflammation may occur, and the respiration may be obstructed; but still the case will want the pathognomonic symptoms of croup.

"Croup, it is stated, frequently succeeds to catarrhal affections, to hooping-cough, to measles, scarlatina, and small-pox." Practice, says M. Guibert, furnishes us with daily examples of this sort. That croup may occasionally be consequential to these disorders, we are not prepared to deny, but we doubt the frequency of the occurrence. Our experience in these diseases has not been trifling, and we do not recollect a single case of genuine croup succeeding to any of them. It is very common for children, during the eruptive stage of measles and scarlatina, particularly if there is much inflammation of the throat, to be suddenly, and without any exacerbation of the symptoms, attacked with a peculiar croaking noise in breathing, which somewhat resembles that which is characteristic of croup. This symptom, however, passes off spontaneously. From dryness and irritation of the larynx, this kind of stridulous breathing very frequently occurs in children; and we have known many instances in which much alarm has been excited, and an active mode of treatment inflicted, when it was perfectly unnecessary. The practical tact of M. Guibert would, we doubt not, prevent him from falling into the error of believing the case to be croup, from the accidental occurrence of this individual symptom.

From the commencement of croup, the voice of the patient is altered; it is hoarse and croaking. There is a painful, burning sensation, and a feeling of constriction in the larynx and trachea; deglutition, however, is not impeded: the respiration gradually becomes laborious and sonorous. Many attempts have been made to define the peculiar noise that is made in breathing, by different comparisons. The practitioner can only become acquainted with it by hearing it, and, when once it has caught his ear, it will not be forgotten. A cough succeeds by paroxysms, which is at first dry, and subsequently attended by expectoration. Fever set in early in this disease; and the general symptoms rapidly increase in severity. The anxiety becomes excessive; the cough more frequent and painful; the patient discharges from the mouth a viscid, frothy fluid, sometimes tinctured with blood, but generally white. This fluid increases in opacity and consistence. The child sometimes throws forth albuminous concretions, resembling a tube, or portions of membrane: their expulsion is not affected without much difficulty and distress, and occasionally vomiting takes place; but, when once this adventitious membrane is discharged, a temporary alleviation of the symptoms almost always follows. At a more advanced period of the disease, the symptoms are still increased in severity, and the unfortunate little patient exhibits a melancholy picture of suffering, which we can rarely alleviate. Respiration is almost entirely impeded; the head is thrown backwards; the anxiety of the child is now at its highest degree; the agitation is extreme. The movements are sudden, irregular, and convulsed. The face becomes of a dark blue colour; the eyes appear unnaturally prominent, are turgid with blood and suffused with tears, and are incessantly rolling in their orbits. The pulse intermits, and disappears entirely under the slightest pressure of the finger. The voice is gradually extinguished, and death soon closes the scene.

The progress of croup is not always regular: an exacerbation of the symptoms frequently occurs towards night; and sometimes, when the death of the child appears inevitable, an unexpected remission takes place. The ordinary duration of croup is from three to six days: very acute cases not unfrequently terminate fatally within twenty-four hours.

The appearances observed upon dissection are—redness of the mucous membrane of the air-passages, an increased secretion of mucus, and layers of membranous concretions, very much resembling those which are produced upon the surface of inflamed serous membranes. Sometimes these adventitious membranes extend as far as the epiglottis, and even line a part of the pharynx. They vary much in form and consistence. Gangrene of the parts affected rarely takes place.

Our limits oblige us to pass over some interesting observations upon the occasional complications of croup.

*Treatment.*—In the first stage, the disease presents but one urgent indication—that of opposing the development and progress of inflammation, by the usual antiphlogistic means. M. Guibert particularly recommends the very frequent use of warm baths, in this as well as in every other case of intense inflammation in children. The directions for the application of leeches appear too indefinite. We are called upon to bleed freely, both locally and generally; but a somewhat more precise guide for the young practitioner would be required, than the option of applying from five to twenty leeches. We are directed to keep the bowels open by enemas; but, for our own parts, we should be more inclined to depend upon calomel and powder of jalap, or scammony.—In the second stage, we have not only to contend with inflammatory action, but we have also to endeavour to procure the expulsion of the mucous or membranous secretion from the bronchiæ and trachea. Some authors have proposed to admit two other indications: to dissolve the false membrane when it is already formed, and to oppose the debility which threatens to take place. Dr. Guibert properly cautions us against the employment of tonics and stimuli in this stage of the disease, notwithstanding they have been very strongly recommended. The debility occurs either from the treatment of the first stage having been carried beyond the point which the powers of the patient are able to bear, or from the local inflammation having arrived at its highest degree of intensity, from the neglect of proper treatment. In the second stage, bleeding will still be required, both locally and externally, in proportion to the severity of the symptoms; and repeated emetics of tartarized antimony are now very strongly indicated. During the efforts of vomiting, the air-passages will be cleared from the morbid secretions which are productive of such danger and distress. The decoction of seneka is considered by our author to be a useful expectorant. There must, however, be considerable difficulty in administering this medicine to children in efficient doses.

Various remedies have been recommended to dissolve the false membrane which is formed,—such as fumigations of volatile alkali, muriate of ammonia, &c. Even the inhalation of chlorine has been advised! These experiments are justly deprecated, as highly improper and dangerous. By many practitioners, the sulphate of potash is highly esteemed as a remedy capable of dissolving the false membrane. Dr. Guibert is apprehensive of its effects in young children, if it be freely administered; and appears sceptical as to its supposed virtues, however judiciously it may be given: he prefers the subcarbonate of potash in small doses. Calcined magnesia is thought to act in the same manner. Of the free and repeated use of calomel, as it is employed in this country, the author speaks rather from report than practical experience; although he admits the efficacy of calomel as a purgative.

To calm the nervous excitement, we are advised to use valerian enemas, and to give the extract of valerian internally. We should ourselves prefer hyoscyamus or conium, and confess we should not hold ourselves justified in wasting time in the exhibition of valerian glysters. Opium is condemned in every form. Blisters are to be applied to the neck, and mustard sinapisms to the feet and legs, in the second stage.

In the third stage, we must exert every effort to prevent suffocation. If emetics are insufficient to throw off the adventitious membrane, vomiting may be excited by the introduction of a feather into the throat. This expedient has been successfully adopted by M. Jurine.

One observation we must add, which may appear trifling, but which is in fact highly important. In every case of inflammation of the larynx or trachea in children, the nurse should be strictly enjoined to watch the patient attentively, and, upon the slightest appearance of mucus in the mouth, to draw it forth by the fingers: the tenacity of the morbid secretion being such, that it is frequently possible to extract long shreds of viscid secretion in this manner; the presence of which has not only been productive of much distress, but has even endangered the life of the patient by suffocation.

The inconveniences resulting from the operation of tracheotomy in the last stages of croup, where death is to be feared from obstructed respiration, have caused it to be generally abandoned, according to Dr. Guibert. In some cases, where every remedy had failed, the operation has been performed in this country with success: we believe, the late Mr. Chevalier performed it more than once, with a fortunate result. If we rescue our patient from the acute attack of croup, much attention will be required, during convalescence, to prevent a relapse.

Eighteen cases of croup are detailed, of which three only terminated successfully.

The second part of the volume is occupied by the consideration of Hooping-Cough. Neither the opinion which the author entertains of the nature of the disease, nor the treatment he recommends, differs materially from that of English physicians.

The third part of the work contains many sound practical observations, although, perhaps, it is somewhat deficient in the charm of novelty. It presents a brief sketch of various inflammatory affections of the throat in children, which bear considerable analogy to croup, and are not unfrequently confounded with it.

To attract attention by new speculations and ingenious hypotheses, is not the aim of the present volume. Dr. Guibert has chosen the less imposing, but certainly more useful course, of simply detailing the results of his own practical experience. By every practitioner the work will be read with interest, although many may find in it nothing more than a confirmation of doctrines they had previously entertained, and the sanction of an able man for a mode of treatment, which does not essentially differ from that generally employed in this country.

### MEDICAL AND PHYSICAL INTELLIGENCE.

